# Atypical angiofibroma in a patient with compromised general health: A case report

**DOI:** 10.1016/j.amsu.2018.09.041

**Published:** 2018-09-28

**Authors:** Artur Cunha Vasconcelos, Silas Antonio Juvencio de Freitas Filho, Vinicius Lima de Almeida, Rafael da Silva Caetano, Julia Tramujas, Luiz Renato Paranhos

**Affiliations:** a Morphological Science, Morgana Potrich College, Mineiros, GO, Brazil; b Post-Graduate Program in Applied Dental Sciences, Bauru School of Dentistry, University of São Paulo (USP), Bauru, SP, Brazil; c School of Dentistry, Federal University of Sergipe, Lagarto, Sergipe, Brazil; d Resident of Oral and Maxillofacial Surgery and Traumatology, Cancer Hospital, Cuiabá, MT, Brazil; e Oral and Maxillofacial Surgeon, Private Practice, Balneário Camboriú, SC, Brazil; f Department of Preventive and Social Dentistry, Federal University of Uberlândia, Uberlândia, MG, Brazil

**Keywords:** Nasopharyngeal angiofibroma, Benign tumor, Soft palate

## Abstract

**Introduction:**

The nasopharyngeal angiofibroma (NA) is a benign tumor that originates from the pterygopalatine fossa and extends to the adjacent anatomical structures and affects frequently young individuals. The basic treatment for NA is surgical resection, but in some cases the tumor is surgically inaccessible.

**Case presentation:**

We describe the case of a 45-year-old male with respiratory difficulty after the appearance of a soft palate lesion. The clinical appearance of NA was not specific.

**Discussion:**

There are still discussions about the best therapeutic strategy and controversies about performing incisional biopsy. To our knowledge, this is the first report of a NA in an adult patient in which the general health conditions prevented the therapeutic approach, besides the extension of the lesion and its complications.

**Conclusion:**

Our case shows that NA may reach high proportions and its involvement in older patients should be considered. In this report, we showed the limitation of the therapeutic strategy for advanced cases of NA.

## Highlights

• A 45-year-old man with soft palate injury and respiratory distress.
• Imaging examination showed involvement of the nasal cavity, nasopharynx and soft palate.
• Initially, an intraoral incisional biopsy was performed under general anesthesia.
• Surprisingly it was a nasopharyngeal angiofibroma.
• General health status and extent of the lesion limited therapeutic intervention.

## 1 Introduction

Angiofibroma is a rare benign fibrovascular tumor that is locally invasive and of unknown etiology; it may affect different parts of the body, including the nasopharynx, which determines the nasopharyngeal angiofibroma (NA) [[1], [2], [3], [4]]. The NA originates from the pterygopalatine fossa and extends to the adjacent anatomical structures [[5], [6], [7]]. In young individuals, this tumor is known as juvenile nasopharyngeal angiofibroma (JNA) [1,4,5]. The NA is more common in men and usually unilateral [[1], [2], [3], [4]]. It is often associated with several signs and symptoms, mainly epistaxis and nasal obstruction [5,[8], [9], [10], [11]].

The diagnosis of NA is based on anamnesis, physical examination, and radiological and morpho-epidemiological findings, and diagnosing this condition is usually challenging [12]. The initial incisional biopsy for diagnosis is controversial due to the high risk of bleeding [5,12]. Different classification methods for NA based on clinical and radiographic findings have been proposed to help defining the surgical approach [10]. The basic treatment for NA is surgical resection, but for cases in which the tumor is surgically inaccessible, radiation therapy has been recommended [5,13]. In addition, cases of extensive NA have been associated with morbidity and mortality [14].

We report a case of NA in a man with an initial lesion in the hard palate, followed by respiratory difficulties. The present case has been reported in accordance with the SCARE criteria [15].

## 2 Case presentation

In March 2016, a 47-year-old male patient was admitted to the Oral and Maxillofacial Surgery and Traumatology service of a public hospital, referred by the medical team with the chief complaint of respiratory difficulty after the appearance of a soft palate lesion. On anamnesis, the patient reported rapid weight loss, drug use for more than 10 years, and the development of a hard palate lesion three years earlier, which was left untreated. In addition, the patient reported the development of a soft palate lesion one month earlier. Intraorally, an extensive mass of mucosa-like color was present in the palate region, which obstructed the oropharynx and consequently caused dyspnea.

**Fig. 1:**
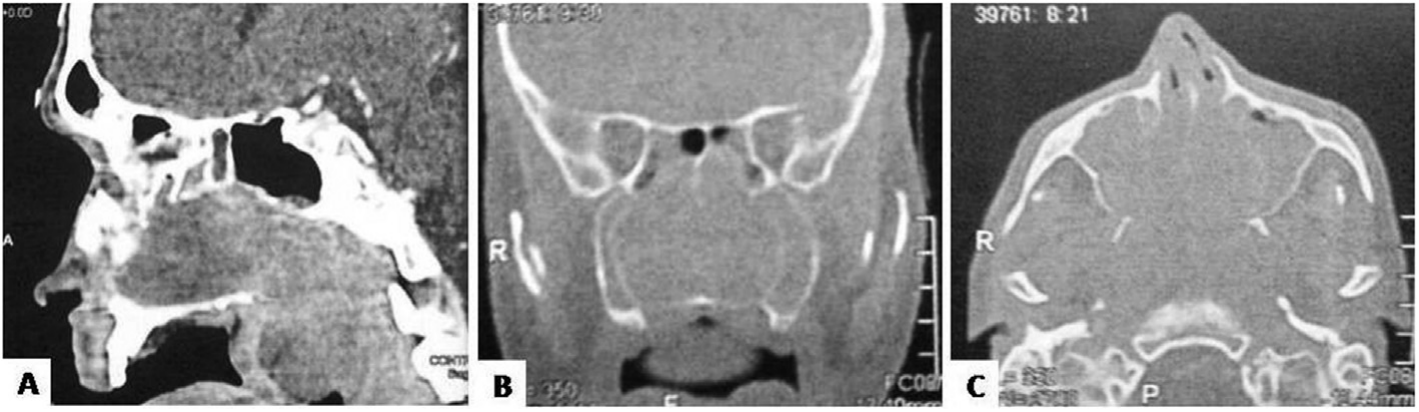
Computed tomography showing sagital (A), coronal (B) and axial (C) views, respectively.

The sagittal computed tomography (CT) image reveals a soft tissue mass in the nasal cavity extending to the posterior portion of the nasopharynx and the lower part of the sphenoid sinus, with lowering of the entire musculature of the soft palate (Fig. 1A). The coronal CT image shows density loss of the septum in the nasal cavity and nasal conchae, maxillary sinus opacification, and lesion extending to the sphenoid cavity floor (Fig. 1B). The axial CT image reveals the soft tissue mass observed in the nasal cavity with opacification of the maxillary sinuses (Fig. 1C).

**Fig. 2:**
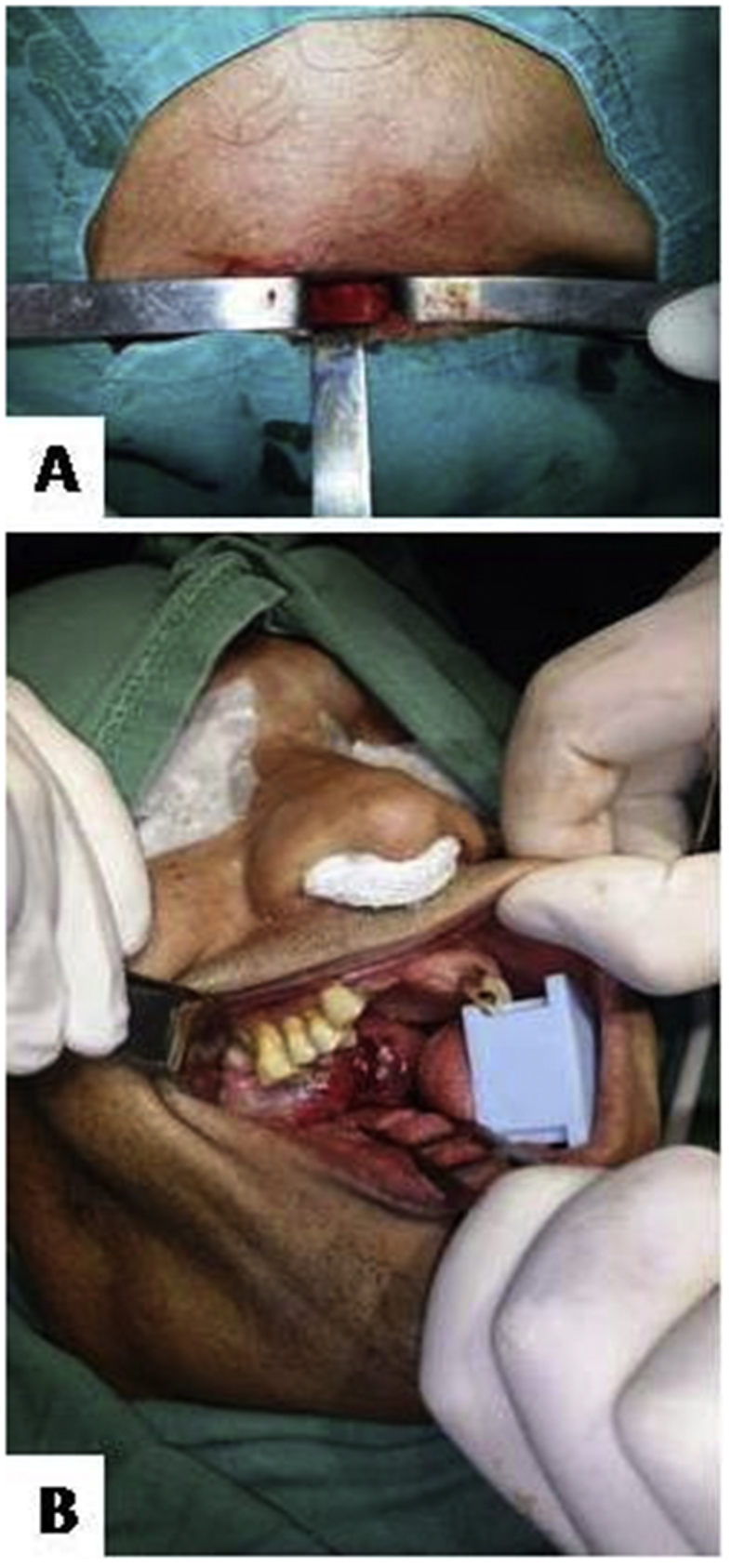
Tracheostomy (A) and intraoral incisional biopsy (B).

**Fig. 3:**
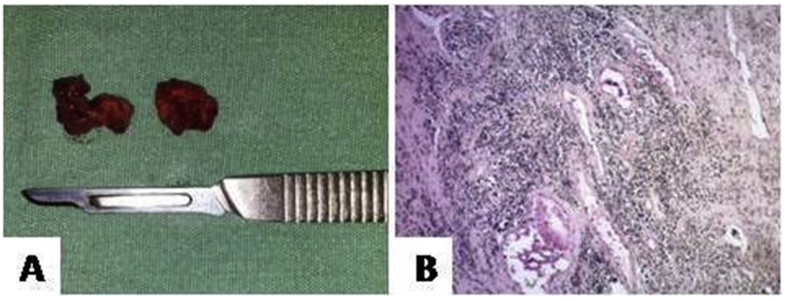
Surgical specimen of the lesion compost by two soft tissue (A) and histopathological analysis – HE staining (B).

After the initial examinations, the patient was submitted to tracheostomy (Fig. 2A) and intraoral incisional biopsy under general anesthesia (Fig. 2B). Intraoperatively, the tumor had a fibrous consistency and showed normal bleeding. The specimen was sent for histopathological analysis (Fig. 3A). Microscopically, the presence of a densely collagenated connective tissue was observed, with numerous blood vessels that were usually of small caliber and sometimes congestive; also, foci of hemosiderosis were visible (Fig. 3B). Based on the microscopic reports, clinical-radiological characteristics and physical examination, the diagnosis of NA was established.

**Fig. 4:**
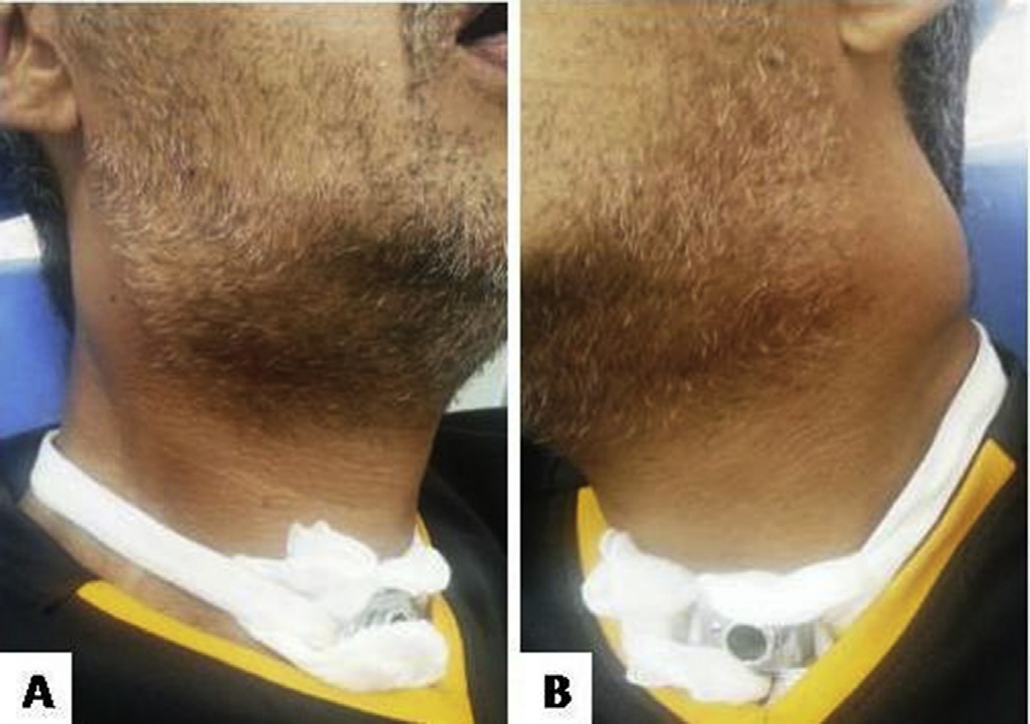
Bilateral swelling in the submandibular and preauricular regions: right (A) and left (B) sides.

The patient was referred for excision of the lesion. However, due to the extension of the lesion and the patient's systemic conditions, the teams of different specialties decided not to perform surgical resection or radiotherapy, opting for embolization. However, as a technology procedure was not available at the time, the patient remained hospitalized awaiting treatment availability. Approximately one month after the incisional biopsy, bilateral swelling in the submandibular region and parapharyngeal regions was noted (Fig. 4A and B). In addition, the patient presented severe headache, difficulty in closing the eyes, dysphagia and dyspnea, trismus, left facial paralysis and episodes of fainting. The patient was under palliative care and died after a few weeks.

## 3 Discussion

Diseases in the nasopharynx may be of different natures, such as benign or malignant neoplasms, and infectious or inflammatory diseases [16]. The NA is a neoplasm that accounts for less than 0.5% of all head and neck tumors [2,5,12,17]. The present case reinforces that the clinical presentation of NA is often associated with respiratory difficulty due to nasal obstruction and maxillary sinusitis. Some cases may present uncontrollable epistaxis [7,10], but our patient did not present this signal. It is recommended, mainly for adolescent males, to include NA in the differential diagnosis of lesions that cause nasal obstruction associated or not with epistaxis [5].

In the case presented, the clinical appearance of NA was not specific. Initially, the diagnostic hypothesis was malignant neoplasm (i.e., adenoid cystic carcinoma). The NA manifests mainly in young men, probably due to increased circulating hormones [1,5]. The patient sought medical care when the lesion was advanced and although it was benign, it invaded the adjacent neurovascular structures, including the oral cavity. According to the classification proposed by Radkowski et al. [18], the tumor in the present case was classified as stage III (A), reaching the base of the skull. From this suspicion, we decided to perform the incisional biopsy. The different degrees of vascularization of this lesion [16] may explain the absence of abnormal bleeding during the diagnostic surgical procedure. In addition, it is worth noting that this case does not represent an extranasopharyngeal angiofibroma [16,17].

Imaging examinations have aided the clinical diagnosis of NA, whereas computed tomography assists in diagnosing bone changes, magnetic resonance imaging evaluates the tumor extension to soft tissues, and angiography estimates the vascular content of the lesion and arterial supply [5,7,9,13]. However, many services have limited resources and, in our case, only computed tomography was performed.

The pathological aspect of NA includes fibrous tissue composed of stellate and spindle cells, with varying degrees of vascularization [1,16]. The stroma may also show variation regarding the density of collagen [1]. Immunohistochemistry has been an auxiliary tool in the diagnosis of these lesions, in which CD34 positivity is observed in blood vessels [2] and the immunoexpression for Ki-67 is restricted to endothelial cells [1]. In our case, the microscopic examination was sufficient to establish the histopathological diagnosis.

There is no consensus on the most appropriate therapeutic strategy for the treatment of NA in relation to morbidity and mortality [9,13]. In addition, the open surgical approach has produced more negative results of recurrence and death [9]. Furthermore, there are discussions on the conduct in advanced cases of NA, especially on either completely removing the lesion or maintaining the remaining tumor [13]. Preoperative embolization has been an option to prevent high blood loss, although it has been more widely used in adolescent patients [7]. In our case, the overall health of the patient was delicate, in addition to significant weight loss, important dysphagia and dyspnea. For this reason, the medical teams decided not to perform interventions due to the risk of death during the surgical removal, and not even submit the patient to radiotherapy because to collateral effects. Unfortunately, the general health was not restored.

## 4 Conclusion

In summary, our case shows that NA may reach high proportions and its involvement in older patients should be considered, even though it is uncommon. In this report, we showed the limitation of the therapeutic strategy for advanced cases of NA in a compromised general health status. We reinforce the need for further unusual and complex cases to be reported, considering the rarity of this tumor.

## Ethical approval

Ethical approval was not required and patient identifying knowledge was not presented in this report.

## Sources of funding

There are no sponsors involved in the study.

## Authors' contribution

All authors contributed signiﬁcantly and in agreement with the content of the article. A.C. Vasconcelos, R.S. Caetano and J. Tramujas participated conducting the patient. S.A.J. de Freitas Filho, V.L. de Almeida and L.R. Paranhos participated in the process of writing of the paper. All authors have read and approved the final version of the manuscript.

## Conflicts of interest

The authors declare that they have no conflicts of interest.

## Research registration number

Not applicable.

## Guarantor

Dr. Luiz Renato Paranhos.

## Consent

Written informed consent was obtained for publication of this case report.

## Appendix A Supplementary data

SCARE Guideline Checklist SCARE Guideline Checklist
